# Multidrug-Resistant *Escherichia coli* Isolate of Chinese Bovine Origin Carrying the *bla*_CTX-M-55_ Gene Located in IS*26*-Mediated Composite Translocatable Units

**DOI:** 10.3390/microorganisms11112795

**Published:** 2023-11-17

**Authors:** Weiwei Wang, Xiaojuan Wei, Safia Arbab, Lingyu Wu, Ningning Lu, Qiqi Zhu, Yubin Bai, Jiyu Zhang

**Affiliations:** 1 Key Laboratory of New Animal Drug Project of Gansu Province, Lanzhou 730050, China; weiweiwang1990@163.com (W.W.); weixiaojuan@caas.cn (X.W.); safiakandhro@gmail.com (S.A.); wly18393716614@163.com (L.W.); luningninglnn@163.com (N.L.); xlzzqq@163.com (Q.Z.); baiyb1011@163.com (Y.B.); 2 Key Laboratory of Veterinary Pharmaceutical Development, Ministry of Agriculture, Lanzhou 730050, China; 3 Lanzhou Institute of Husbandry and Pharmaceutical Sciences, Chinese Academy of Agricultural Sciences, Lanzhou 730050, China

**Keywords:** *bla*_CTX-M-55_, IS*26*, transmission, *E. coli*, translocatable units (TUs)

## Abstract

Elevated detection rates of the *bla*_CTX-M-55_ gene in animals have been reported as a result of antibiotic misuse in clinics. To investigate the horizontal transfer mechanism of *bla*_CTX-M-55_ and its associated mobile genetic elements (MGEs), we isolated 318 nonrepetitive strains of *Escherichia coli* (*E. coli*) from bovine samples in Xinjiang and Gansu provinces, China. All *E. coli* strains were screened for the *CTX-M-55* gene using PCR. The complete genomic data were sequenced using the PacBio triplet sequencing platform and corrected using the Illumina data platform. The genetic environment of the plasmids carrying the resistance *bla*_CTX-M-55_ gene was mapped using the software Easyfig2.2.3 for comparison. The results showed that all *bla*_CTX-M-55_-positive strains were resistant to multiple antibiotics. Five strains of *Escherichia coli* carry the *bla*_CTX-M-55_ gene, which is adjacent to other resistance genes and is located on the IncHI2-type plasmid. Four of the five *bla*_CTX-M-55_-harbor strains carried translocatable units (TUs). All the donor bacteria carrying the *bla*_CTX-M-55_ genes could transfer horizontally to the recipient (*E. coli* J53 Azr). This study demonstrates that the transmission of *bla*_CTX-M-55_ is localized on IS*26*-flanked composite transposons. The cotransmission and prevalence of *bla*_CTX-M-55_ with other MDR resistance genes on epidemic plasmids require enhanced monitoring and control.

## 1. Introduction

Antimicrobial resistance, particularly multidrug resistance, has become one of the greatest threats to global health today. *E. coli* is one of the most common nosocomial and community-acquired Gram-negative pathogens that is resistant to third- and fourth-generation cephalosporins [1,2,3].

The extended-spectrum β-lactamase (ESBL) family in *E. coli* is widespread and prevails, in which *CTX-M* is by far the most prevalent drug resistance gene [4,5]. Today, more than 230 *bla*_CTX-M_ variants are documented in GenBank. In China, the main prevalent drug resistance genes are *bla*_CTX-M-9_ and *bla*_CTX-M-1_ [6]. Of these, the *CTX-M-55* ESBL-positive rate is already overgrown in the clinic [7]. Increasing reports indicate the transmission of the *bla*_CTX-M_ gene alone or its cotransmission with other ARGs in humans, animals, and waterfowl [8,9,10,11]. Recently, *CTX-M-55*-positive *E. coli* strains isolated from cattle feces were widely reported in China, Canada, South Korea, and France [8,12,13,14], suggesting that *bla*_CTX-M_ is an important drug resistance gene in cattle from food animal sources. Furthermore, the cotransmission of *CTX-M-55* with different resistance genes has also been reported [9]. Therefore, the genetic environment of *bla*_CTX-M-55_ needs to be explored to analyze the evolutionary and transmission mechanisms of drug resistance.

As in previous reports, *bla*_CTX-M_ is seldom on chromosomes but mainly localized on plasmids, such as IncHI2, IncFIA, and IncFIB [15,16,17,18]. Plasmid-encoded CTX-M enzymes have a robust horizontal transmission capability between humans and food animals, implying that they can be transmitted directly from animal products to humans, thereby ultimately causing a potential contamination risk of drug resistance [4]. Additionally, mobile genetic elements (MGEs) located on plasmids can widely spread antibiotic resistance genes (ARGs) [19]. The insertion sequence (IS), one of the MGEs, can significantly facilitate the transfer of ARGs between chromosomes and plasmids [20]. It has been reported that IS (such as IS*26*, IS*Ecp1*, and IS*CR1*) is commonly found to be adjacent to *bla*_CTX-M_, which plays a vital role in promoting the horizontal transmission of *bla*_CTX-M_ genes [18,21,22]. MGEs on plasmids have been a worrying feature, leading to their global spread and evolution [4,23].

Although the inappropriate use of antibiotics contributes to the persistence and spread of antibiotic resistance, there is limited information available on this topic from developing countries. The misuse and abuse of cephalosporins in clinics have resulted in the high rates of detection of the *bla*_CTX-M-55_ gene in food animals. As a result, there is an increased risk of transmission from food animals to humans caused by *bla*_CTX-M-55_-bearing plasmids. To investigate if the occurrence of cephalosporin resistance has also increased with the high frequency of *bla*_CTX−M_ and the horizontal transfer frequency of *bla*_CTX-M_ in food animal isolates in recent years, *E. coli* isolates of bovine origin collected during 2018 for cephalosporin resistance and plasmid-mediated cephalosporin resistance genes were screened, and the genetic environment of the positive strain was analyzed to explore the contribution of certain MGEs to the horizontal transfer of the *bla*_CTX-M-55_ gene.

## 2. Materials and Methods

### 2.1. Strain Isolation and Identification

The study collected 232 nonrepetitive fecal samples from cattle at a farm (*n* = 73) in the provinces of Xinjiang Province, China, and two farms (*n* = 159) in the provinces of Gansu Province, China, between August and December 2018. All samples were collected in sterile containers, transported to the laboratory at 6 °C ± 2 °C, and processed immediately for further assays.

All 318 *E. coli* isolates were selected on MacConkey agar plates (Huankai, China), and the species were identified with MALDI-TOF-MS and 16S rRNA sequencing using universal primers (see Table S1). Positive strains were screened for the presence of *CTX-M-55* using PCR [24].

### 2.2. Antimicrobial Susceptibility Testing

Antimicrobial susceptibility testing of strains toward 15 different antimicrobials was determined with broth microdilution using breakpoints specified by Clinical & Laboratory Standards Institute (CLSI) M100 guidelines (http://clsi.org) (accessed on 10 October 2020). Briefly, bacterial colonies were selected and cultured overnight in 1 mL of MH broth. The following day, a 10 μL aliquot of the bacterial solution was absorbed and re-inoculated into 1 mL of fresh MH broth, followed by incubation at 37 °C, to obtain a bacterial concentration of approximately 1 × 10^8^ CFU/mL. A 180 μL volume of MH broth was added into the first column of a 96-well plate and an additional 100 μL was also added into each subsequent well. After adding 20 μL of the tested antibiotic stock solution to the first column, serial dilutions were performed using the double-dilution methodology. Column eleven served as the negative control, while column twelve served as the positive control. Bacteria were diluted one hundred times with fresh MH broth to obtain a final concentration of approximately 1 × 10^6^ CFU/mL before adding it (100 μL) into antibiotics (200 μL). Finally, the plate was incubated at medium culture temperature (37 °C). The following antibiotics were used for antimicrobial susceptibility testing: cefotaxime (CTX), ceftazidime (CAZ), cefalotin (KF), ceftriaxone (CRO), tetracycline (TE), ampicillin (AMP), amikacin (AK), ciprofloxacin (CIP), doxycycline (DO), fosfomycin (FOT), kanamycin (K), chloramphenicol (C), sulfamethoxazole–trimethoprim (SXT), gentamicin (GN), and aztreonam (ATM). *E. coli* ATCC25922 was used as the quality control strain.

### 2.3. Conjugation Assay and Determination of Conjugation Frequency

Conjugation was performed using *E. coli* J53 Az^r^ as the recipient strains as previously described and conducted with solid mating on a filter [25] (Whatman, Maidstone, UK). The donor–recipient ratio was 1:1 using Mueller–Hinton medium (MHA, Huankai, Guangzhou, China) supplemented with cefotaxime (2 μg/mL) and sodium azide (200 μg/mL) as the selective medium. The transconjugants were analyzed via PCR (*bla_CTX-M-1_* primers, see Table S1), Sanger sequencing, and antimicrobial susceptibility testing to compare their consistency with the donor strain. Transfer frequencies were calculated as the number of transconjugants per recipient.

### 2.4. Whole-Genome Sequencing and Bioinformatics Analysis of CTX-M E. coli Producer

Five isolates identified as positive for *bla*_CTX−M−55_ using PCR and Sanger sequencing were further characterized with whole-genome sequencing. The total genomic DNA of five strains was extracted with the SDS method [26]. The harvested DNA was detected using agarose gel electrophoresis and quantified using a Qubit^®^ 2.0 Fluorometer (Thermo Scientific, Waltham, MA, USA). Libraries for single-molecule real-time (SMRT) sequencing were constructed with an insert size of 10 kb using the SMRT bell TM Template kit, version 1.0. Briefly, the process involved fragmenting and concentrating DNA, repairing DNA damage and ends, preparing blunt ligation reactions, purifying SMRTbell Templates with 0.45× AMPure PB Beads, size selection using the BluePippin System, and repairing DNA damage after size selection. Finally, the library quality was assessed on a Qubit^®^ 2.0 Fluorometer (Thermo Scientific), and the insert fragment size was detected using Agilent 2100 (Agilent Technologies, Santa Clara, CA, USA). The whole genomes of selected strains were sequenced using the PacBio Sequel I platform and Illumina NovaSeq6000 at Beijing Novogene Bioinformatics Technology Co., Ltd. (Beijing, China).

Gene prediction and annotation were performed using the RAST server (https://rast.nmpdr.org/) (accessed on 6 May 2021) and the BLAST program of NCBI (https://blast.ncbi.nlm.nih.gov/Blast.cgi) (accessed on 6 May 2021). The replicon of the plasmid was analyzed using PlasmidFinder 2.1 (https://cge.cbs.dtu.dk/services/PlasmidFinder/) (accessed on 7 May 2021). The antibiotic resistance genes were identified using CARD data (https://card.mcmaster.ca/) (accessed on 10 May 2021). Clonal analysis was assessed using MLST 2.0 (https://cge.food.dtu.dk/services/MLST/) (accessed on 10 May 2021). Serotyping analysis was assessed using SerotypeFinder 2.0 (https://cge.food.dtu.dk/services/SerotypeFinder/) (accessed on 10 May 2021). The comparative analysis and plasmid map were generated with Easyfig2.2.3 and BRIG [27,28]. Specific primers were designed for further analysis using Primer Primer v.5.0 (see Supplementary Table S1). Targeted sequences, also known as IS*26*-mediated composite transposons, were detected using reverse PCR, and amplified products were confirmed using Sanger sequencing [29].

### 2.5. Plasmid Stability

Plasmid stability was determined according to Lv’s [30]. Briefly, without antibiotics, these strains were cultured continuously in daily refreshed LB broth with 1000-fold dilution for 15 days to detect their stability. Within each 5-day period, 20 colonies were randomly selected and confirmed using the PCR amplification of *bla*_CTX-M-55_ (*bla*_CTX-M-1_ primers, see Table S1). The plasmid retention rate was further calculated as mentioned above over a period of 15 days.

### 2.6. Statistical Analysis

Mean values and standard deviations were calculated using SPSS 17.0 version software. Student’s *t* test was used to evaluate differences between means, with a significant probability at a *p* value of <0.05.

### 2.7. Nucleotide Sequence Accession Numbers

The nucleotide sequences of pXJ5.2-plas1, pXJ6.1-plas1, pXJ55-plas1, p ZYB39-plas2, and pZYB62-plas1 have been uploaded in GenBank under accession numbers CP074355, CP074357, CP098230, CP098236, and CP074367, respectively.

## 3. Results

### 3.1. Characteristics of CTX-M-Positive E. coli

The presence of the *bla*_CTX-M-55_ gene was confirmed in five *E. coli* isolates (1.57%, 5/318), of which three (3/5) were from dairy cattle and two (2/5) were from beef cattle. These five isolates were recovered from beef and dairy cattle in three different farms located in two provinces (Gansu and Xinjiang). The MIC results showed that all five isolates were multidrug-resistant (MDR) and resistant to at least nine antibiotics. All five isolates showed resistance to KF, CTX, CAZ, CRO, AMP, C, GN, and ATM (Table 1). 

### 3.2. Genetic Environment of the bla_CTX-M-55_-Harboring IncHI2 Plasmid

The genetic contexts of five *bla*_CTX-M-55_-positive isolates were performed using WGS. The five *bla*_CTX-M-55_-positive plasmids ranged in size from 209 to 230 kb and contained IncHI2 replicons (*n* = 5) (Table 2). The MLST showed that five *CTX-M-65*-positive strains belong to ST5044, ST155, ST6345, ST58, and unknown type, respectively (Table 1). Moreover, these five strains were categorized under different serotypes (Table 1). 

For pXJ5.2-plas1, the complete genome contained a circular 209 kb plasmid with GC content of 46.0%. The multidrug-resistant region (MRR) region of *bla*_CTX-M-55_ exhibited 100% sequence identity (coverage: 100%) with pST45-1 (CP050754.1) carried by *Salmonella* sp. and pL725-unnamed2 (CP036204.1) carried by *E. coli* (Figure S1). In addition, the *bla*_CTX-M-55_-carrying IncHI2 part of pXJ5.2-plas1 showed a high similarity with the plasmid pVb0499 (MF627445.1), with 89% coverage and 100% identity (Figure S1). The sizes of pXJ55-plas1, pZYB39-plas2, and pZYB62-plas1 were almost 230 kb, with GC contents of 47.0%, 47.0%, and 46.0%, respectively (Table 2). The MRR on the plasmid (region of 15,018 bp) was homologous to pAMSH1 (CP030940.1) (89% coverage and 100% identity), pE105-4 (CP072315.1) (100% coverage and 99.98% identity), p2016062-242 (CP090540.1) (100% coverage and 99.97% identity), and p2017028-250 (CP090546.1) (100% coverage and 99.96% identity) (Figure S2). *Bla*_CTX-M-55_ was flanked by IS*26* and IS*1380*, also demonstrating that the IS*26* composite transposons IS*26*–*bla*_CTX-M-55_–IS*Ecp1*–IS*26* were conducive to transmission between different species (IS*Ecp1* and IS*26*).

**Figure 1:**
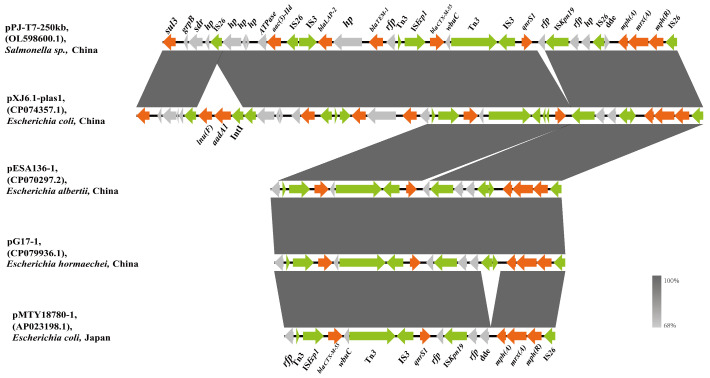
Sequence alignment of *bla*_CTX-M-55-_positive IncHI2 plasmids of pXJ6.1-plas1. Green arrows represent mobile elements; the orange arrows represent resistance genes; gray arrows represent other features.

pXJ6.1-plas1 is a circular 267 kb plasmid of type IncHI2 with 47% GC content. *bla*_CTX-M-55_ was located on the pXJ6.1-plas1 plasmid within 33,246 bp and exhibited 100% sequence identity (coverage: 93%) with the plasmid of pPJ-T7-250 kb carried by *Salmonella* sp. (Figure 1), except that pXJ6.1-plas1 possessed a 4.3 kb insertion (IS*26*- *lun(F)*-*aadA1*-orf2-hp-IS*26*). This corresponding region was also found in pESA136-1 (CP070297.2) of *E. albertii* from China, pG17-1 (CP079936.1) of *E. hormaechei* from China, and pMTY18780-1 (AP023198.1) of *E. coli* from Japan, indicating that the transmission of *bla*_CTX-M-55_ harboring this structure occurred among *E. coli*, *Salmonella* sp., *E. albertii,* and *E. hormaechei*.

**Figure 2:**
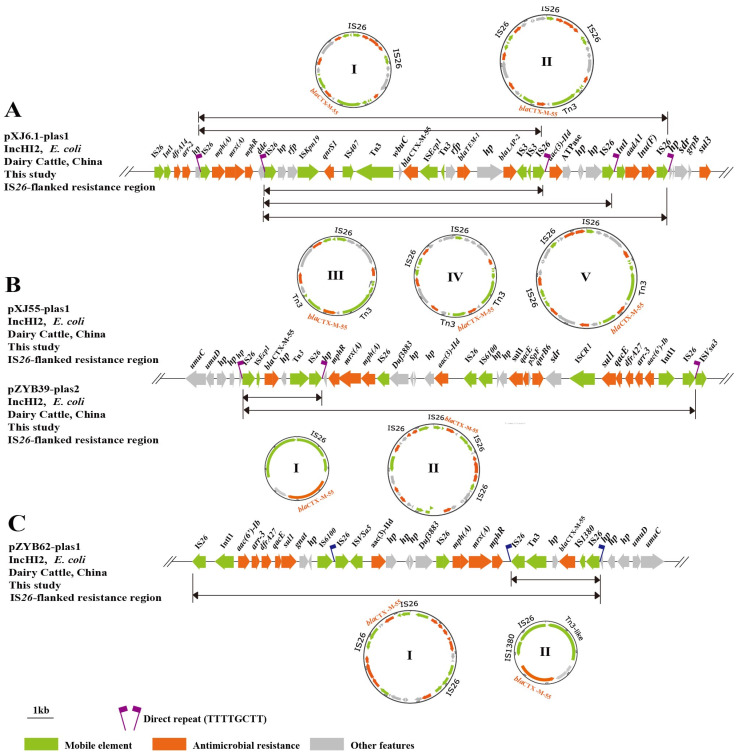
Genetic and molecular analysis of the *bla*_CTX-M-55_-positive plasmids. (**A**–**C**) Schematic representation of the circular forms obtained from pXJ6.1-plas1, pXJ55-plas1, pZYB39-plas2, pXJ55-plas1 and pZYB62-plas1 using PCR and sequencing.

IS*26*-mediated composite transposons were amplified from pXJ6.1-plas1, pXJ55-plas1, pZYB39-plas2, and pZYB62-plas1 using reverse PCR [31] (Figure S3). Four of the five *bla*_CTX-M-55_-harboring strains carried a total of 11 circular intermediates. Particularly, pXJ6.1-plas1 contained five cyclic intermediates (Figure 2). 

**Figure 3:**
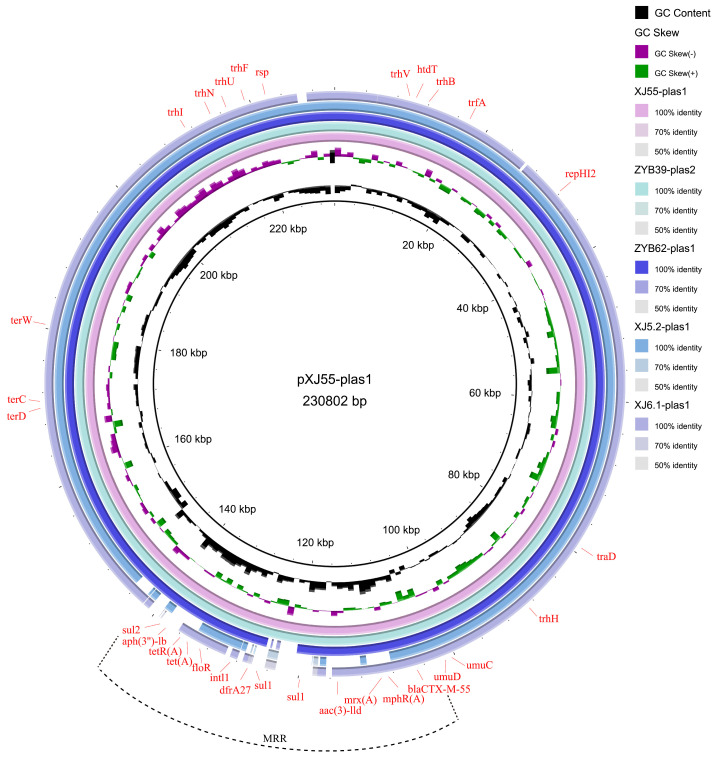
Characteristics of the complete nucleotide sequence of *CTX-M-55* pXJ55-plas1 identified in this study. A comparison of pXJ55-plas1 with four other *CTX-M-55* plasmids, i.e., pZYB39-plas2, pZYB62-plas1, pXJ5.2-plas1, and pXJ6.1-plas1, was also performed in this study.

To observe the similarity between the five plasmids harboring *bla*_CTX-M-55_, the alignment of the complete nucleic acid sequence of these five plasmids was generated using BRIG. The backbone region of pXJ55-plas1 was almost identical to that of pZYB39-plas2, pZYB62-plas1, pXJ5.2-plas1, and pXJ6.1-plas1 (Figure 3). Except for the backbone region, the major differences among the five plasmids were concentrated in the MRR and TraC (transfer protein) and ParB (N-terminal domain-containing protein) of the backbone.

In addition to the plasmid backbone region, plasmid pXJ55-plas1 harbored a 44.6 kb MRR containing 11 ARGs (*bla_CTX−M−55_*, *aac(3)-III*, *sul1*, qnrB6, arr-3, *aac(6)-Ib*, *floR*, *tet(A)*, *tet(R)*, *aph(3”)-Ib,* and *bla*_TEM-1_) interspersed with different complete or truncated insertion sequences and transposons (IS*26*, IS*Ecp1*, Tn*3*, IS*CR1*, and Int*1*) (Figure 3). The MRR containing *bla*_CTX−M−55_ of pXJ55-plas1 was highly heterogeneous compared with the other four plasmids.

### 3.3. Transconjugative Frequencies of Plasmids Carrying bla_CTX-M-55_

As a donor, the *CTX-M*-positive strain was transferred to recipient *E. coli* J53 according to the conjugation assay. Five plasmids carrying *bla*_CTX−M−55_ were all successfully transferred into the recipient strain. The conjugation frequencies of IncHI2 plasmids are listed in Table 3 as transconjugants per recipient. The conjugation frequencies of IncHI2 plasmids were 10^−5^~10^−8^ per recipient.

### 3.4. Plasmid Stability

**Figure 4:**
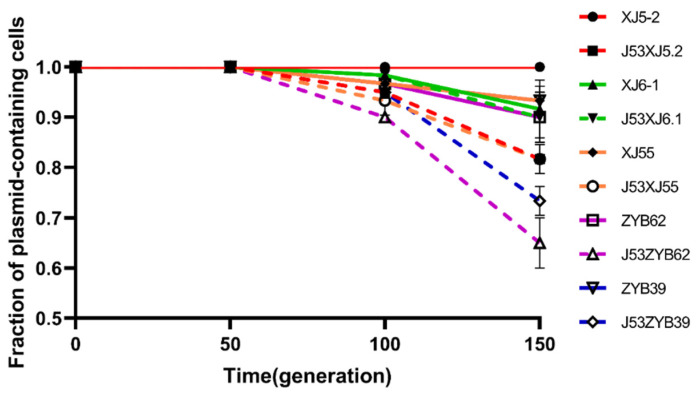
Stability of *bla*_CTX-M-55_-positive and transconjugants of *E. coli* J53. Error bars stand for standard deviations (*n* = 3).

The evaluation of plasmid stability was performed at 37 °C in the absence of antibiotic selective pressure. pXJ6.1-plas1, pXJ5.2-plas1, pXJ55-plas1, pZYB39-plas2, pZYB62-plas1, and their transconjugants of *E. coli* J53 harboring IncHI2 plasmids were stable within 10 consecutive days (retention ≥ 0.9) but were gradually lost from *E. coli* J53 transconjugants after 10 days (Figure 4).

## 4. Discussion

The detection of *CTX-M*-positive isolates from Western China has been conducted by our group, revealing that the most prevalent genotype of the *CTX-M* gene in Gansu beef cattle is *CTX-M-55* (27.9%, 36/129) [32]. However, the detection rate of *CTX-M-55* in dairy cattle from Xinjiang Province was 11.1% (12/108). Extended-spectrum cephalosporin (ESC) resistance is a common prevalence in livestock [33], especially in *E. coli* strains producing *CTX-M*-type ESBLs, which has become a worrying issue. In the United States, bovine-derived ESC-resistant (ESC-R) *E. coli* resistance rates are as high as 95% [34], while in Asia, they range from 1% to 33% [35,36,37,38]. All of these data suggest that *E. coli* serves as a reservoir for *CTX* resistance genes, and the spread of its resistance is a matter of serious concern.

In this study, five multidrug-resistant *bla_C_*_TX-M-55_-positive isolates were all resistant to cefotaxime. Furthermore, they displayed a wide spectrum of antibiotic resistance to additional common antibiotics in the clinical and breeding industry, such as β-lactams, aminoglycosides, and tetracycline. High-frequency therapeutic failures in the treatment of ESBL-producing microorganisms can be expected as these resistant strains spread.

The results of STs showed that the clones in this study did not involve high-risk clones (Table 1), such as ST131 or ST69 [39]. However, ST58, which is a major extraintestinal pathogenic (causing urinary tract infection) *E. coli* lineage in humans, was found in clones carrying *bla*_CTX-M-55_ in our study and has also been reported in numerous countries [40,41,42]. Furthermore, since XJ5.2 is serotype O45 with some pathogenicity and resistance, its transmission should be provided extra attention.

IS*26*, belonging to the IS*6* family, has a simple organization and generates an 8 bp DR upon insertion [43]. In this cointegration process, the second formed IS copy is directly oriented as the original copy and flanked by DR sequences [44]. In the process of study, we found that the *bla*_CTX-M-55_ genetic environment of pXJ55-plas1 and pZYB39-plas2 was flanked by directly oriented copies of IS*26* with 8 bp DR sequences (TTTTGCTG). However, IS*26* and potential IS*26*-mediated transposons do not necessarily generate the flanking DR, and intramolecular transposition will lead to the loss of the flanking repeat [43]. Notably, pXJ6.1-plas1 is the plasmid containing the most TUs and the highest number of copies of IS*26*. This may be related to the increased activity and multiple copy number of IS*26* in the plasmid [45]. IS*26* can enhance the expression of the *bla*_CTX-M-55_ gene, and its presence could explain the ease with which this gene is spreading among bacteria and different species. The TU contains a single IS*26* copy and neighboring DNA, which plays a vital role in the diffusion of ARGs in Gram-negative bacteria to form complex resistance regions [46]. No TU was detected for pXJ5.2-plas1. Only *bla_CTX-M_*_-55_ was flanked by a single copy of IS*26* in p*CTX-M-55*-XJ5.2, suggesting that it is difficult to create a circular form because of a single copy of IS*26* [47,48]. It is now widely accepted that any gene(s) bound by two directly oriented copies of IS26 should be able to be formed into a TU via either an IS26-mediated process or via homologous recombination in a recombination-proficient host. Overall, the TU on plasmids harbor the *bla*_CTX-M-55_ gene and other ARGs through IS*26*-mediated translocation events.

In contrast to the conserved backbones, the *bla*_CTX−M−55_-containing MRRs of the five plasmids analyzed in our study were heterogeneous. The acquisition or deletion of resistance determinants mediated mobile genetic elements, and recombination predominantly contributed to heterogeneity [21]. In addition, the genetic environment of *bla*_CTX-M-55_ in the plasmid analyzed here is similar to those reported from different countries (Figure 1, Figures S1 and S2), suggesting that this genetic environment was conducive to the transmission of genes carrying multiple ARGs, including the *bla*_CTX-M-55_ gene. From the observations in this study, *bla*_CTX-M-55_ is localized on the IncHI2-type plasmids. IncHI2, along with IncI1, IncF, and IncA/C2 type plasmids, are commonly considered the main epidemic plasmids carrying *bla*_CTX-M-55_ for transmission [10,49]. The IncHI2-type plasmid on the XJ5.2 strain carrying *bla*_CTX-M-55_ remained a genetically stable plasmid under continuous culture without any antibiotic selection pressure for 15 consecutive days (with a retention of 100%) (Figure 4), which contributed to horizontal spreading of the plasmid. These results suggest that the diverse and flexible spread of the *bla*_CTX-M-55_ resistance gene is related to heterogeneous MRR and a special type of plasmid, IncHI2.

## 5. Conclusions

Five strains carrying *CTX-M-55* have been isolated from the Chinese provinces of Xinjiang and Gansu. The genetic context of the *bla*_CTX-M-55_ resistance gene was examined using the Illumina and PacBio platforms. IS*26*-mediated transposons were identified using WGS analysis and reverse PCR method in *E. coli* isolated from beef and dairy cattle in China, suggesting a potential for transmission along animal lines to humans. Five plasmids carrying *bla*_CTX-M-55_ also show some features of transfer capability and a relative stability. The cotransmission and prevalence of *bla*_CTX-M-55_ with other MDR resistance genes on epidemic plasmids require enhanced monitoring and control.

## Figures and Tables

**Table 1:** Background information and characteristics of *bla*_CTX-M-55_-carrying *E. coli* isolates.

Strain	Province	Cattle	MLST	Serotype	Resistance Profiles
XJ5.2	Xinjiang	Dairy	ST5044	O45:H45	KF/CTX/CAZ/CRO/C/K/AMP/GN/ATM/
XJ6.1	Xinjiang	Dairy	NT	O23:H24	KF/CTX/CAZ/CRO/TE/C/K/AMP/GN/DO/ATM/SXT
XJ55	Xinjiang	Dairy	ST155	O88:H25	KF/CTX/CAZ/CRO/TE/C/AMP/GN/ATM/SXT
ZYB39	Gansu	Beef	ST6345	O83:H7	KF/CTX/CAZ/CRO/TE/C/AMP/GN/DO/ATM/SXT
ZYB62	Gansu	Beef	ST58	O8:H21	KF/CTX/CAZ/CRO/TE/C/AMP/GN/ATM/SXT

NT, unknown-type; resistances common to all isolates are KF/CTX/CAZ/CRO. CTX, cefotaxime; CAZ, ceftazidime; KF, cefalotin; CRO, ceftriaxone; TE, tetracycline; K, kanamycin; AMP, ampicillin; GN, gentamicin; DO, doxycycline; C, chloramphenicol; SXT, sulphamethoxazole–trimethoprim; and ATM, aztreonam.

**Table 2:** Characterization of plasmids carrying *bla*_CTX-M-55_.

Plasmids	Size (kb)	Replicon Type	Resistance Genes	Number of TUs
pXJ5.2_1	209	IncHI2	*bla*_CTX-M-55_ */dfrA14/aadA5/sul2/floR*	NE
pXJ6.1_1	267	IncHI2	*bla*_CTX-M-55_/*dfrA14/qnrS1/bla*_ΔTEM_*/lap/aac(3’)-III/aadA1/lnu(F)/sul3/tetA/tetR/floR/aph(3″)-Ib*	5
pXJ55_1	230	IncHI2	*bla*_CTX-M-55_ */aac(3’)-III/sul1/qnrB6/aac(6′)-Ib/floR/tetA/tetR/strB/aph(3″)-Ib/bla*_TEM-1_	2
pZYB39_2	230	IncHI2	*bla*_CTX-M-55_ */aac(3’)-IId/sul1/qacE/qnrB6/dfrA27/arr-3/aac(6′)-Ib/floR/tetA/strB/aph(3″)-Ib/bla*_TEM-1_	2
pZYB62_1	226	IncHI2	*bla*_CTX-M-55_ */aac(6′)-Ib/arr-3/dfrA27/qacE/sul1/floR/tetA/strB/aph(3″)/bla*_TEM-1_	2

NE, non-existent.

**Table 3:** Transconjugative frequencies of *bla*_CTX-M-55_-positive isolates.

*E. coli* Strain (Transconjugants)	Transconjugative Frequencies ^a^
	pJ53*CTX-M* ^b^
J53XJ5.2	4.7 × 10^−5^
J53XJ6.1	3.6 × 10^−8^
J53XJ55	1.2 × 10^−6^
J53ZYB39	1.8 × 10^−8^
J53ZYB62	4.3 × 10^−8^

^a^ The experiment was repeated three times. ^b^ Cefotaxime was used as the selection pressure.

## Data Availability

We confirm that all supporting data, code, and protocols have been provided within the article or through Supplementary Data Files. Five collections of whole-genome sequences of strains were used in the study, and accession numbers and associated metadata can be found in the Nucleotide Sequence Accession Number Section as indicated. The whole-genome sequence was uploaded and registered in the NCBI database with accession numbers: CP074354 (Escherichia XJ5.2-chr1), CP074355 (Escherichia XJ5.2-plas1), CP074356 (Escherichia XJ6.1-chr1), CP074357 (Escherichia XJ6.1-plas1), CP074358 (Escherichia XJ6.1-plas2), CP098229 (Escherichia XJ55-chr1), CP098230 (Escherichia XJ55-plas1), CP098234 (Escherichia ZYB39-chr1), CP098235 (Escherichia ZYB39-plas1), CP098236 (Escherichia ZYB39-plas2), CP074366 (Escherichia ZYB62-chr1), CP074367 (Escherichia ZYB62-plas1), CP074368 (Escherichia ZYB62-plas2), and CP074369 (Escherichia ZYB62-plas3).

## Supplementary Materials

The following supporting information can be downloaded at: https://www.mdpi.com/article/10.3390/microorganisms11112795/s1, Figure S1: Sequence alignment of *bla*_CTX-M-55-_positive IncHI2 plasmids of pXJ5.2. GeneBank accession numbers are shown in parentheses; Figure S2: Sequence alignment of *bla*_CTX-M-55_-positive IncHI2 plasmids of pXJ55-plas1, pZYB39-plas2, and pZYB62-plas1. GeneBank accession numbers are shown in parentheses; Figure S3: Detection of circular intermediates in plasmids using reverse PCR. From left to right, the first column is a marker, and the second to sixth columns are circular forms I, II, III, IV, and V of pXJ6.1-plas1 (matching with Figure 2); the seventh to eighth columns are circular forms I and II of pZYB39-plas2; the ninth to tenth columns are circular forms I and II of pXJ55-plas1; the eleventh to twelfth columns are circular forms I and II of pZYB62-plas1. Table S1: Primers for strain identification and detection of the circular intermediate form.
