# Supplementary material for: Multidrug-Resistant Escherichia coli Isolate of Chinese Bovine Origin Carrying the blaCTX-M-55 Gene Located in IS26-Mediated Composite Translocatable Units
Source: Microorganisms. 2023 Nov 17;11(11):2795. doi: 10.3390/microorganisms11112795 (PMC10673294; doi:10.3390/microorganisms11112795)
Supplement: Supplementary file 1 [file microorganisms-11-02795-s001.zip › microorganisms-2685782-supplementary.pdf]

Table S1. Primers for strain identification and detecting the circular intermediate form.

Primers	Sequence (5'-3')	Product length
16s	F:GAGCGGATAACAATTTCACACAGG	500bp
	R: CGCCAGGGTTTTCCCAGTCACGAC	
bla$_{CTX\text{-}M\text{-}1}$	F: AACCGTCACGCTGTTGTTAG	766bp
	R: TTGAGGCTGGGTGAAGTAAG	
XJ6-1-I	P1: CGTTTTTGCTCTCTTACGGTTGCG	3867bp
	P2: GCGTTGAGAATGGGAGAGACTGAGC	
XJ6-1-II	P1: AAGGAAGGACCCTGTGTCTCAACTC	3818bp
	P2: CGCCTGGCAACTGTGAAAGC	
XJ6-1-III	P1: CATTATCACAGCGGAACTCA	3221bp
	P2: AAACTGACTGTCTTTGTATCACTCT	
XJ6-1-IV	P1: AGCCAACTGCTGCTGCGT	1739bp
	P2: GCTCAAAGAAATTGTATTCATAGGT	
XJ6-1-V	P1: TTGTTATGACTGTTTTTTTGGGGT	3422bp
	P2: ATAACTTCAGGCTCCAGTACCG	
XJ55-I	P1: CTTATGCAACATCGTATTATTCTCC	2762bp
	P2: GATTTAACAGATTCGGTTCGCT	
XJ55-II	P1: GCAAGGTTTCGGTCTCCA	3349bp
	P2: GGCTCGCTTCTGCTTTCAC	
ZYB39-I	P1: TTCGCCTTACCCGTCTTTTA	2844bp
	P2: ATTTAACAGATTCGGTTCGCTT	
ZYB39-II	P1: ATGAAGTGGTTCGCATCCTCG	3207bp
	P2: TGGGCTCGCTTCTGCTTTC	
ZYB62-I	P1: ATCAATGCCACACCCAGTCTGC	2616bp
	P2: CAACATCGTATTATTCTCCCCG	
ZYB62-II	P1: CTCAAGTCCGATGACGAGGTAC	3095bp
	P2: CCGAACAGGCTTATGTCCACT	

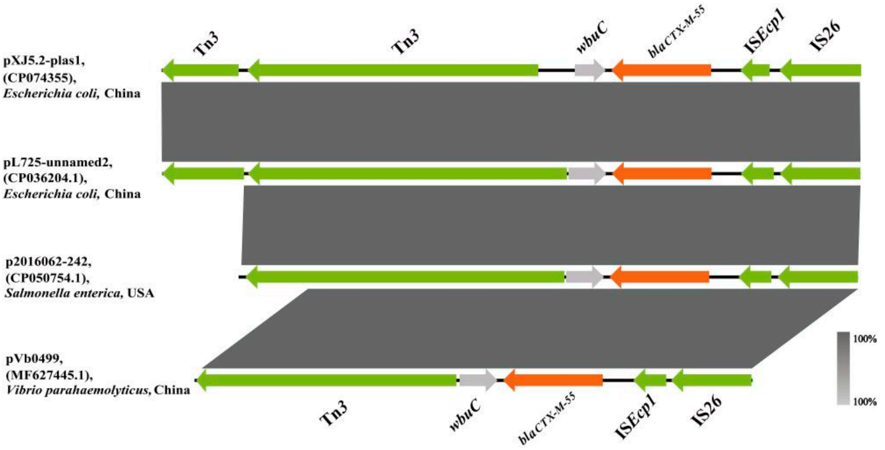

Figure S1. Sequence alignment of *bla*$_{CTX\text{-}M\text{-}55}$-positive IncHI2 plasmids of pXJ5.2-plas1.

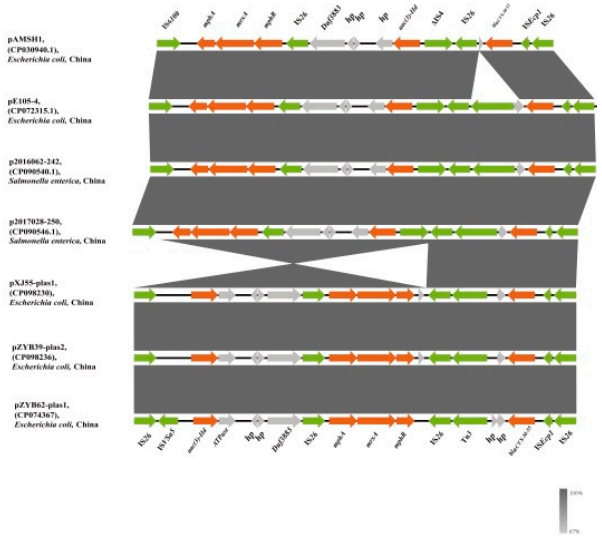

Figure S2. Sequence alignment of bla$_{CTX-M-55}$-positive IncHI2 plasmids of pXJ55-plas1, pZYB39-plas2, and pZYB62-plas1.

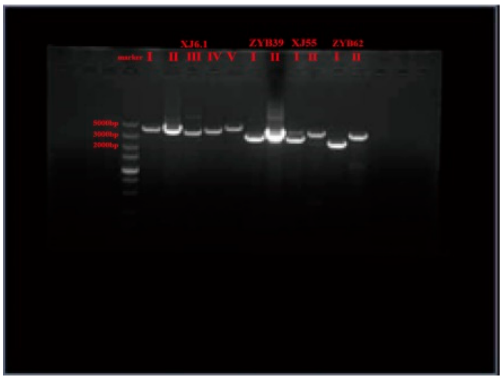

Figure S3. Detection of circular intermediates in plasmids using reverse PCR. From left to right, the first column is marker, and the second to sixth columns are circular forms I, II, III, IV, and V of pXJ6.1-plas1 (matching with Fig.2); the seventh to eighth are circular forms I, II of pZYB39-plas2; the ninth to tenth are circular forms I, II of pXJ55-plas1; the eleventh to twelfth are circular forms I, II of pZYB62-plas1.
